# Engaging with immigrant students’ voices in the school environment: an analysis of policy documents through school websites

**DOI:** 10.1186/s12889-024-18427-8

**Published:** 2024-04-19

**Authors:** Maritta Välimäki, Kirsi Hipp, Faye Acton, Angelika Echsel, Ioan-Alexandru Grădinaru, Katrin Hahn-Laudenberg, Christina Schulze, Elisabeth Stefanek, Georg Spiel, Niamh O’Brien

**Affiliations:** 1 Department of Nursing Science, Faculty of Medicine, University of Turku, Turku, Finland; 2 School of Public Health, University of Helsinki, Helsinki, Finland; 3 School of Health and Social Services, Häme University of Applied Sciences, Hämeenlinna, Finland; 4 Faculty of Health, Medicine and Social Care, Anglia Ruskin University, Chelmsford, UK; 5 School of Health Professions; Institute of Occupational Therapy, Zurich University of Applied Sciences, Winterthur, Switzerland; 6 Faculty of Philosophy and Social-Political Sciences, “Al. I. Cuza” University of Lași, Lași, Romania; 7 Faculty of Education, Leipzig University, Leipzig, Germany; 8 Pro Mente Forschung (Pro Mente Research), Linz, Austria; 9 Pro Mente: Kinder Jugend Familie (Pro Mente: Children Youth Family), Klagenfurt am Wörthersee, Austria

## Abstract

**Background:**

For students to feel happy and supported in school, it is important that their views are taken seriously and integrated into school policies. However, limited information is available how the voices of immigrant students are considered in European school contexts. This study generated evidence from written documents to ascertain how student voice practices are described at school websites.

**Methods:**

Between 2 March and 8 April 2021, we reviewed the policy documents publicly available on school websites. The schools located in areas of high immigration in six European countries: Austria, England, Finland, Germany, Romania, and Switzerland. The READ approach was used to guide the steps in the document analysis in the context of policy studies (1) ready the materials, 2) data extraction, 3) data analysis, 4) distil the findings). A combination of qualitative and quantitative approaches with descriptive statistics (n, %, Mean, SD, range) was used for analysis.

**Results:**

A total of 412 documents (305 schools) were extracted. Based on reviewing school websites, reviewers’strongly agreed’ in seven documents (2%) that information related to seeking student voices could be easily found. On the contrary, in 247 documents (60%), reviewers strongly indicated that information related to seeking student voices was missing. No clear characteristics could be specified to identify those schools were hearing students’ voices is well documented. The most common documents including statements related to student voice were anti-bullying or violence prevention strategies (75/412) and mission statements (72/412).

**Conclusions:**

Our document analysis based on publicly accessible school websites suggest that student voices are less frequently described in school written policy documents. Our findings provide a baseline to further monitor activities, not only at school level but also to any governmental and local authorities whose intention is to serve the public and openly share their values and practices with community members. A deeper understanding is further needed about how listening to student voices is realized in daily school practices.

**Supplementary Information:**

The online version contains supplementary material available at 10.1186/s12889-024-18427-8.

## Background

Today there are some 1 billion migrants globally, about 1 in 8 of the global population, whose well-being is strongly related to the social determinants including education [1]. To have a positive school experience, immigrant children and adolescence, regardless of the reasons for immigrating, need to feel heard within the school environment [2]. Opportunities to be heard can be realised through enabling these students to have an influence on outcomes in a democratic way [3–5] and implementing student voice initiatives into school policies and practices [6, 7]. Proposed by Shallcross, Robinson, Pace and Tamoutseli [8], paramount to student voices is being heard and being able to express their own views. Having a sense of belonging, being heard and valued, being involved in joint decisions, and seeing the impact from their actions all contributed to secondary school students’ sense of agency [9].

Identifying the presence of students’ voices within schools policy documents is important for many reasons. First, the practice of student voices is an indicator of a democratic school environment [10]. There should therefore be better understanding ‘how and why certain policies come to be developed in particular contexts, by who, for whom, based on what assumptions and with what effect’ [11]. Second, student experiences of active participation in decision-making processes at school can diminish the negative effect of ethnic victimization on immigrant youth’s self-esteem, and thus increase their satisfaction with school and academic expectations [12]. Third, democratization of school culture and policies through student voice initiatives within a context of marginalized students, i.e. immigrant students who have experienced racism and trauma, can add to a positive learning environment for all [13]. In addition, by offering voice to all students, schools can contribute to overall positive health and well-being as well as developing strategies towards a more tolerant society and respect for others [14].

Although student voices practice is valued in school contexts, its realization has been doubted for many reasons. Studies have found unequal participation among students at school because of their socioeconomic status [15, 16]. In Australia, Black [15] reported that marginalised students’ participation in school policy was rare and they had no real opportunities to make changes due to the under-representation of marginalised student representatives on the school council [15]. Mager and Nowak [16] reported based on the synthesization of 32 empirical studies that despite students’ participation in school councils, temporary working groups, and different decision-making environments, positive effects of student participation was moderate or low [17]. Although school-level policies are promising in principle, they often lack sufficient financing, program quality, and effective coordination [18]. Further, despite attempts to develop approaches, interventions and an environment that encourages listening to students [19–22], many have little opportunities to meaningfully engage in decisions related to policies, programs, and services [23]. Further, practices in the transparency of policy documents may vary in different countries and schools, which may indicate country-specific institutionalized ideas, rationales, and discursive practices, not only on school evaluation, but also on school accountability or public information [24]. School-based interventions to hear student voices, have also been criticized for their limited ability to target the full range of multi-faceted problems especially in youth refugees [25]. The absence of student voices in public health approaches at schools has also been recognised [26].

It is still unknown how student voices are captured within school policy documents, a practice that has been suggested as poorly developed in the school context [27]. In this study, we specifically focused on immigrant students including asylum seekers and refugees who are often considered marginalized [21]. Marginalised students without access to existing community and school-based supports are less likely to engage with school-based activities including student voice initiatives [28]. For those students traumatized by past experiences [29], student voice practices have been found to provide a space to be heard while schools can become more supportive and inclusive environments [30]. Consequently, understanding how student voice practices as demonstrated in their own policy documents, could provide an insight into how this adds to a positive learning environment. To determine the effectiveness of policies regardless of their contents in the future, it is also important to evaluate the actors and processes involved in policy development and implementation, as investigating the evidence of these variables could be a worthwhile endeavor for future research [31]. To do so, more understanding of specific characters of schools related to realization of student voices is also needed. If variations based on specific characteristics exist between the volume and content of the retrieved information across schools, countries, and geographical areas, the differences could infer that there are country-specific institutionalized ideas, rationales and discursive practices, not only on the school evaluation, but also on the school accountability or public information within this wider European region [24]. This could further lead to greater diversity in how the provision of listening to students is conducted. With this in mind, we hypothesized that engaging students in school initiatives such as student voice work, should clearly be identified in school policy documents [7, 8]. We also assumed that there will be discrepancy between schools and countries about how student voice work is documented and represented in different schools and locations based on the specific characteristics of the schools [30].

We therefore aimed to describe how student voices have been addressed in European schools as evidenced from websites of schools located in high immigrant areas in six European countries: Austria, England, Finland, Germany, Romania, and Switzerland. To our knowledge, it thus fills a gap in the current research and provides an added value compared to the existing knowledgebase. More specifically, our research aim was three-fold. First, we described, the extent to which hearing ‘student voices’ in schools in areas of high immigration can be identified in publicly available policy documents on the school websites. Second, we identified the specific characteristics of the schools where student voices are well identified based on the policy documents. And third, we described how student voices are represented in school policy and other documents on publicly available school websites.

## Methods

### Design

Policy document analysis [32] was used to review how student voices are heard and implemented in European schools in areas of high immigration, as evidenced from their websites. Policy document analysis was selected because it is a useful method for understanding policy content across time and geographies and how information and ideas are presented formally [33]. The rationale for the document analysis is revealed from a constructionist approach where the analytical focus on policy documents is informed by the value of those documents and the interactive research questions guiding the inquiry [34]. The approach fits with our study because according to mainstream policy studies, policies are understood as an interaction of values, interests and resources guided through institutions and mediated through politics [35], which are further featured prominently in policy texts [36]. In this study, the READ approach, i.e. ready the materials, data extraction, data analysis, and distil the findings, was used to guide the steps in the document analysis to collect documents and generate information in the context of policy studies [33].

### Settings

Data collection was conducted from school districts across six European countries: Austria, England, Finland, Germany, Romania, and Switzerland. Study countries represent different European geographical areas with diverse economic and cultural contexts and educational systems. Populations ranged from 5.5 to 84 million people with a diverse range of languages spoken, between one and four in each country. Compulsory education started at age 4 to 6 years and ended at ages 15 to 18 years depending on the country. To ensure that the schools selected for our research were likely to have students with different backgrounds, we therefore decided that we would focus our sample on living areas of high immigration. Schools were funded by municipalities or local government. Half of the countries had legislation relating to student voice initiatives (Table 1). A description of the school system in the countries included in the study is described in Additional file 1.

**Table 1:** Characteristics of the participant countries and their educational system

	**Countries**					
	Austria	England	Finland	Germany	Romania	Switzerland
**Population (MM**^**a**^**)**	9.0	56.3	5.5	84.0	19.1	8.7
**Official languages**	German	English	Finnish, Swedish	German	Romanian	German, French, Italian, Romansh
**Population born abroad**	15%	14%	7.6%	16.55%	9.8%	30.8%
**Student age**^**b**^** (years)**	6–15	4–18	6–16	6–16	6–18	4–15
**Funding**	State, federal states, municipalities	Local authorities, not-for-profit academy trusts, foundation bodies	Municipalities	Federal states (staff) & Municipalities (buildings)	State, local authorities, other sources (sponsorships or donations)	Municipalities
**Management of education**	Centralized	National curriculum, local authority or independent	Centralized	Decentralized (federal states level)	Partially decentralized	Decentralized
**Legislation of student voice initiatives**	Yes	No	Yes	Yes	No	No

^a^MM = Million

^b^Compulsory education

### Eligibility criteria

Internal policy documents from school websites were selected as data for this study as school policy documentation provides information about the schools official discourses [37] representing ‘social facts’ [38]. In this study, policy documents were referred to as ‘formal or informal legislative or regulatory action, statements of intent, or guides to action issued by governments or organizations’ [39]. These documents were used to analyse policy processes, as an assessment of multi-sectoral planning process as evidence for valuing student voices in school policy documents [33].

The documents were defined as written organization-wide strategies addressing key issues, principles, and values of the school. These included mission statements, policies, guidelines, rules, or other written documents publicly available on the school websites. The inclusion criteria for schools were: schools providing education to students up to and including those aged 18 years; public websites easily accessible by the public (no access codes required) and the website content in written format in the country’s main national language. The schools were located in areas of high immigration defined in national documentation. Further, if the information led to other websites or sources, only the primary source was extracted. We excluded any social media sources or unofficial websites on the school website or external links leading to other national level websites outside the specific school website.

### Ready the materials

In each country, the process of data extraction was undertaken between 2 March and 8 April 2021. Schools located in areas of high immigration were identified using Google web engineer, government level websites, or relevant documents. As publicly available information should be easily available on the school website, no longer than 20 min were spent on each school website. However, the time limit was only indicative aiming to show that information available to a public audience should be easy-to-access, without specific insight knowledge of the structure or content of the website. The websites were screened for eligibility (document selection). If more than one document per school was found, all documents were extracted separately by the local reviewer. Our target sample in each country was 50 policy documents, which was guided by data adequacy concerning about the ability of the extracted data to provide a rich and nuanced account of the phenomenon studied based on data saturation [40].

### Data extraction

All 50 documents identified in each country were saved in a local data management system for further analysis. The characteristics of the schools were extracted with a specific tool designed for the study by the authors, including geographical location, school type (public, private, other), number of students, possible specialties in student composition, and age range of students in each school were extracted. In addition, the document type, name, aim and target group of the document were identified.

To describe the extent that hearing ‘student voices’ at schools in areas of high immigration can be identified within publicly available policy documents on school websites (the research question 1), a combination of qualitative and quantitative approaches were used. This exploratory sequential analysis method began with qualitative data extraction and analysis phase, which built to the subsequent quantitative phase [41]. Combining qualitative and quantitative approaches was useful in our study as it helped us to gain a more complete understanding of the issues in the data [42]. First, specific topics were identified for consideration in the written policy documents as clear statements about student voice initiatives or activities related to student voices. This approach focused on text (words, sentences, paragraph) used in the targeted documents, which were identified and extracted in the specific Excel table designed for the study.

Second, to show ‘to what extent’ student voices were heard at schools in areas of high immigration, a quantitative approach was used. This aimed to ensure that documents were examined and interpreted similarly in different contexts and cultural locations. This was done by forming a structured data extraction tool for the quantitative data [43] to reduce, classify and synthetize raw data [44]. The data extraction tool included 8 items and the representative author/s from each country rated the following items:

1. The general question: Student voice had been sought in the process of developing the document (1 = strongly disagree – 7 = strongly agree).

Specific questions related to evidence were asked about student voices in the retrieved documents. If the evidence was clear the reviewer assigned the value ‘1’, if it was not clear the reviewer assigned the value ‘0’. This evidence related to:

2) Specific methods were used to seek student views in developing the document. (1 = yes, 0 = no).
3) Students participated in a document development group (1 = yes, 0 = no).
4) Students feedback was sought in reviewing the document (1 = yes, 0 = no).
5) The documents cited or referred to existing literature (1 = yes, 0 = no).
6) The document evidenced how student voice(s) informed the development of the document (1 = yes, 0 = no).
7) Student diversity was reflected in the document (1 = yes, 0 = no).
8) The document described how student voices were considered or ensured in school practices and processes (1 = yes, 0 = no).
9) Any methods used by the school to seek student input, including consultation, interviews or surveys in the document development (1 = yes, no = 0).

The content of each document was then reviewed using the data extraction tool. Questions raised during the data extraction and analysis process were discussed with the first two authors. Data from each country were further combined and checked by the same authors to ensure consistency of the analysis. In the case of any missing data or unclear coding, the questions were addressed with the country representatives.

### Data analysis

Characteristics of the documents and schools, and numerical data extracted from the written policy documents were analysed and described using descriptive statistics (n, %, Mean, SD, range). To identify schools where hearing student voices were rated was ‘high’, the dataset was further re-coded by combining scores 7 and 6 so that either rank 6 or 7 (agree or strongly agree) represents a ‘highly ranked ‘student voice school’. Further, characteristics and possible differences between schools and the documents in each country were descripted by simple crosstabulation in Tables. The data were analysed using SPSS version 27 (IBM Corp). To illustrate the results, examples of methods addressing student voices were provided from specific school documents with the school’s identification number (ID).

## Results

### Characteristics of the documents and schools

A total of 412 documents from 305 schools met the inclusion criteria (Table 2). The number of documents analysed per country varied from 49 (Switzerland) to 110 (Finland). The most common documents were anti-bullying or violence prevention strategies (75/412) and mission statements (72/412). The target group of the document was stated in 217 documents as follows: the wider public (*n* = 106), students (*n* = 53), parents/carers (*n* = 5) and school staff (*n* = 3). From all the documents, fifty were intended for more than one target group.

**Table 2:** Types of documents analysed in six European countries

		**Countries**					
		Austria	England	Finland	Germany	Romania	Switzerland
**Type of document**	N (%)	N (%)	N (%)	N (%)	N (%)	N (%)	N (%)
Anti-bullying or violence prevention strategy	75 (18%)	3 (6%)	50 (82%)	3 (3%)	10 (11%)	3 (6%)	6 (12%)
Mission statement	72 (17%)	25 (48%)	0 (0%)	5 (4%)	11 (13%)	10 (19%)	21 (43%)
School rules	50 (12%)	15 (29%)	0 (0%)	21 (19%)	9 (10%)	5 (10%)	0 (0%)
Inclusion or diversity strategy	45 (11%)	2 (4%)	4 (7%)	5 (4%)	33 (37%)	0 (0%)	1 (2%)
School curricula	38 (9%)	3 (6%)	0 (0%)	28 (26%)	7 (8%)	0 (0%)	0 (0%)
Combination	23 (6%)	0 (0%)	0 (0%)	0 (0%)	17 (19%)	6 (12%)	0 (0%)
Other	109 (27%)	4 (7%)	7 (11%)	48 (44%)	2 (2%)	27 (53%)	21 (43%)
Total	412	52	61	110	89	51	49

School characteristics were based on data from 305 schools in the six countries, representing 89 cities/municipalities in 154 geographical areas. Typically, the schools were public (*N* = 295) although a small number of ‘other’ schools were represented including private or religious schools. Student numbers varied from 10 (England) to 1,700 (Switzerland). Variation in student age was evident, ranging from 2 years (England) to 22 years (Romania) as some schools provided education to younger children and those with special education needs (Table 3). Existing student voices literature was referred to in 30 documents (7%).

**Table 3:** Characteristics of the schools

		**Countries**					
		Austria	England	Finland	Germany	Romania	Switzerland
**Characteristics**	N	n	n	n	n	n	n
**Number of schools**	305	52	50	50	50	53	50
**Cities/municipalities**	89	1	1	13	2	42	30
**Geographic location**	154	17	2	50	12	42	31
**School type**							
Public	295	52	46	45	49	53	50
Private	7	0	4	2	1	0	0
Other	3	0	0	3	0	0	0
**Students, n range**	10–1700	194–500	10–313	200–1159	28–1500	687–1476	150–1700
**School including diversity groups**^**a**^**, n (%)**	55 (18%)	16 (31%)	8 (16%)	28 (56%)	2 (4%)	1 (2%)	0 (0%)
**Age range (years)**	2–22	10–14	2–19	5–19	6–18	3–22	5–16

^a^As far as explicitly mentioned on the website

#### The extent that ‘student voice’ is heard at schools in areas of high immigration based on publicly available policy documents on school websites (Research question 1)

**Fig. 1:**
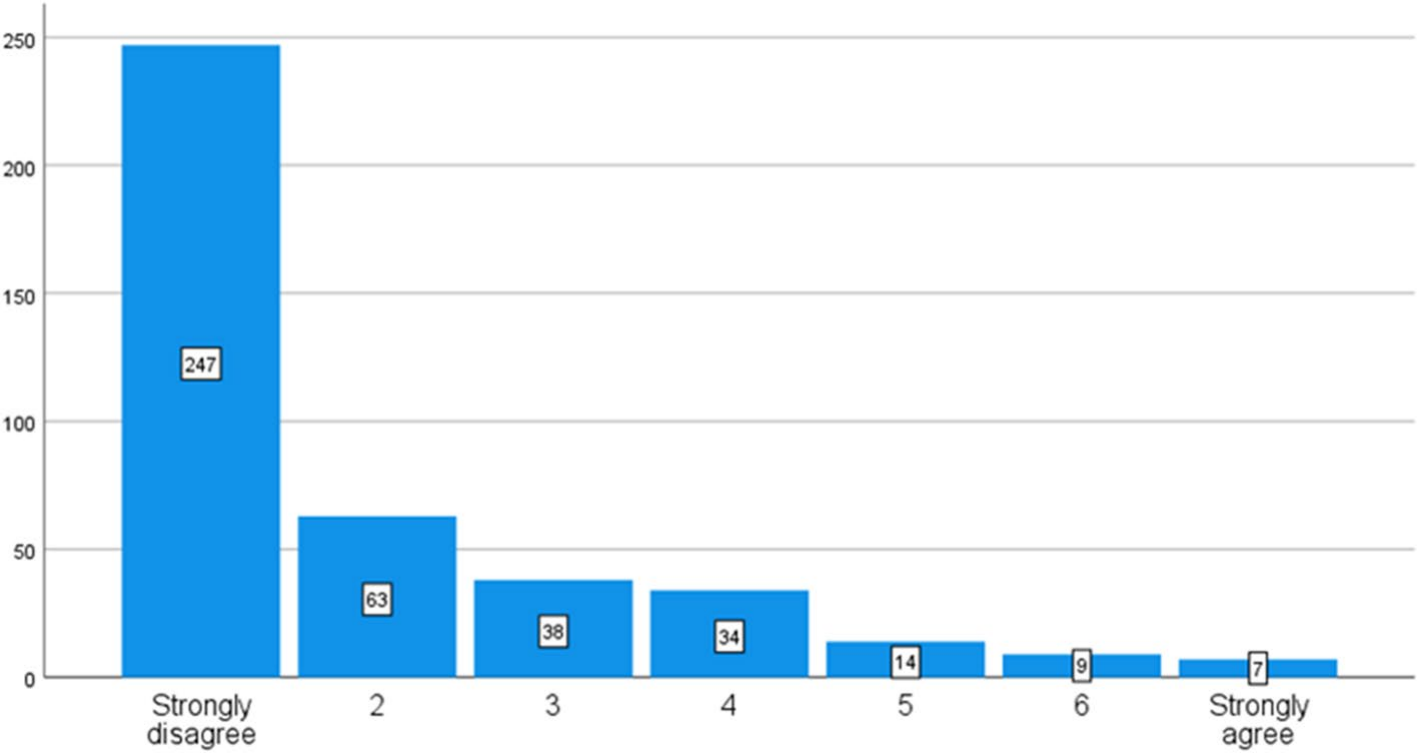
The extent of how hearing student voices were documented during the development process of the documents (*N* = 412)

The reviewers were asked to respond on the extent that student voices were sought in the process of developing the document based on the written information in the documents on the school websites (the 7-point scale, 1 = strongly disagree, 7 = strongly agree). Of the 412 documents, reviewers ‘strongly agreed’ in seven documents (2%). On the contrary, in 247 documents (60%), reviewers strongly indicated based on reviewing school websites that information related to seeking student voices was missing (Fig. 1.)

The processes of how the schools described listening to student voices within the documents was evidenced in 124 (30%) documents, while student diversity was reflected in 120 (29%) documents.

#### Characteristics of the highly ranked schools hearing student voices (Research question 2)

Across the dataset (412 documents), 16 documents were rated ‘high’ by the reviewers (scores 7 or 6) meaning that students voices are well described in the school documents. These documents came from schools in England (*n* = 6), Romania (*n* = 5), Finland (*n* = 2), Switzerland (*n* = 2) and Germany (*n* = 1) (Table 3). Of the 16 schools, 15 were government funded. The size of these schools varied from 81 to 1,159 students (M = 378), and they did not describe any specialties in student composition. Almost half (*n* = 7) of these schools reported strategies relating to anti-bullying or violence prevention (Table 4).

**Table 4:** Characteristics of the highly rated ‘student voice schools’ (*N* = 16) and methods of how student voices are heard based on the documents on the school websites

Document ID	Country	Number of students	Type of document	Consultation, interview or survey	Participation in development group	Feedback for the draft	Citation of existing literature	Use of gathered information	Student voice processes	Student diversity
69	England	140	Anti-bullying	X	X	-	X	X	X	X
74	England	266	School vision	X	X	X	-	X	X	-
85	England	81	Anti-bullying	X	X	X	X	X	-	-
88	England	186	Anti-bullying	X	-	-	X	X	X	-
90	England	240	Anti-bullying	X	X	X	-	X	X	X
112	England	270	Anti-bullying	X	X	X	-	X	X	-
118	Finland	1159	School curricula	X	X	-	-	-	X	X
151	Finland	630	Annual report	X	X	-	-	-	-	-
309	Germany	-	Inclusion/ diversity	X	-	-	-	-	-	X
327	Romania	-	Project report	-	-	-	-	-	X	-
330	Romania	-	Project report	-	-	-	-	-	X	-
346	Romania	-	Blog article	X	-	-	-	X	X	-
352	Romania	-	School rules	X	X	-	-	-	-	-
359	Romania	-	Anti-bullying	X	X	-	-	-	-	-
394	Switzerland	360	Student parliament protocol	X	-	-	-	-	-	-
411	Switzerland	450	Anti-bullying	X	X	X	-	X	-	X

#### Representation of student voices in school policy and other documents (Research question 3)

Specific methods used to address student voices was reported in 105 documents representing 25% of 412 documents. Student voices were mostly heard using student consultation of their views, opinions, interviews or surveys (*n* = 70, 17% out of 412 documents). Students were also encouraged to participate in document development (*n* = 55, 13% out of 412 documents). Twenty-three documents (6%) included information about how student voices were used to inform the document development. Twelve (3%) documents reported that student feedback was sought for its draft.

The most frequently reported methods for hearing student voices at ‘highly ranked’ schools (14/16) were identified in the documents like consultation, involvement, interviews, and surveys. Examples in the data included topics of planning upcoming school events. Students were also consulted on their views about bullying or preparing surveys to understand school bullying. Students were able to share experiences through writing school leaflets about their school year and be involved in developing school curricula. Further, students were offered an opportunity to give new ideas in the class. Examples identified in the written documents can be found below (ID represent verification of the specific document).

*Ihe information about the graduation ceremony was passed on to the classes by all present. So far, the classes have not come up with any ideas related to the program. Therefore, we discussed that everyone should think about who in their class is good at something (music, dance, giving speeches, etc.) so that individual students can be addressed specifically. (ID 394)*

*Pupils and their guardians have participated in making the curriculum, especially related to values, work culture, and the meaning of cultures and languages. Involvement was enabled for example by surveys and discussions on the school open day and parents' evenings. (ID 118)*

Ten out of 16 highly ranked documents demonstrated that students had participated in the document development group. Students had ‘hands-on’ involvement by writing anti-bullying policies and documents, diversity strategies, or they drafted a list of school visions and values. In one document, student union boards participated in developing school rules and decision-making on how student behavior and working skills were evaluated, as well as contributing to the final production of the document.

*The focus group has met frequently to distill the outcomes of all the consultations and drafted a list of vision and values. (ID 74)*

Feedback on the draft of any school document was reported in 5 of the 16 documents. For example, in one school, students working as anti-bullying ambassadors annually reviewed the anti-bullying policy. Student voices had also been sought about drafting the school vision. One document was written by students themselves.

*This policy is based on DfE guidance “Preventing and Tackling Bullying” July 2017 and supporting documents. (ID 85)*

Use of gathered information was illustrated in half of the documents (8/16). It was reported that student views had supported the development of school values and helped the document development group to see how the school vision would look in practice. It was also noted that student feedback was useful for informing future provision of anti-bullying policies and how to regularly seek student views in the future.

*There is an on-going consultation process which includes: --- Regularly checking the views of elected pupil representatives within the schools e.g. pupil leaders, student council and pupil voice. (ID 69)*

Nine documents out of 16 included a description of student voices processes implemented in the school. These included specific responsibility roles, such as elected student representatives and anti-bullying ambassadors. Student voices were discussed in relation to safe climate and opportunity to communicate and express opinions. Two documents instructed students on how they can communicate their thoughts and concerns or devised creative methods for students to work together in groups to find solutions to bullying while one document illustrated that student participation in developing, planning, and evaluating the working culture was promoted by "own teacher's moments", development discussions, peer supporter action and a student union board. Documents described that student voice was addressed as outlined in national policies.

*Anti-Bullying Ambassadors provide peer support to encourage students to report any kinds of bullying behaviour that they have been in receipt of or have witnessed (ID 90)*

*Students should create films/games that reflect episodes of bullying (either in real life or online). Students are advised to work together in groups and find solutions to the problems described. Their films/games will be uploaded on educational platform. (ID 330)*

In addition, five documents (5/16) reflected student diversity by describing bullying in different groups in relation to race, sexual orientation or ‘being different’ in any other way. Also, documents stated commitment to promoting community cohesion and implementing necessary actions related to ethnicity, religion, or culture, or described methods to promote students lingual and cultural identity. Diversity was also found intrinsic to the content of strategy documents.

*This shows that the society here at school is not yet ready to accept all people. We find this fact sad. With this letter, we would like to draw attention to the grievances. (A letter from a class ID 411)*

*To promote the development of lingual and cultural identity, students are offered, if possible, their own language teaching in cooperation with city of XXX. Pupils' own languages and cultures are dealt in the subjects and projects to which they naturally are related. (ID 118)*

## Discussion

### Main findings

Our study aimed to describe the extent to which hearing ‘student voices’ at schools can be identified within publicly available policy documents on school websites. Despite the global emphasis to strengthen student voices in terms of having their say in wider society [45–48], we found a limited number of publicly available documents on school websites to clearly indicate that student views and preferences were sought.

In our data, the most common method identified to seek student voices in school documents was student consultation, followed by student participation in developing the document. On the contrary, students’ feedback on school documents was less often used according to the documents found on school websites. Indeed, some experts have defined that listening to student voices is a spectrum ranging from expression and consultation on the lower end to activism and leadership on the higher end [49]. Reflecting our finding to the idea of a spectrum on student voices may indicate that student consultation in our data still represents the lowest level of students’ participation type. Our results raise questions as to whether student voices in general are linked to reality or rhetoric or if the result is due to a lack of reporting on how student voices are described and captured in the documents. More studies are therefore still needed to understand the role of student voices in school policy documents.

Previous studies have reported that there are specific characteristics of the schools which are relevant for the realization of participation and student voices [48, 49]. For example, difference between volume and content across schools, countries, and geographical areas could infer that there are country-specific institutionalized ideas, rationales and discursive practices, not only on the school evaluation, but also on the school accountability or public information [18]. We therefore assumed that it is possible to identify specific characteristics to describe the schools where student voices are highly represented in school public documents. Contrary to these results, no clear characteristics related to the ‘student voice schools’ could be identified based on country, size of the school, students, specialties in student composition or existing strategies relating to anti-bullying or violence prevention. Interestingly, Maxwell and Granlund [50] found differences between two countries, Scotland and Sweden, in terms of how student participation was expressed in written education policy documents. The authors proposed that these differences could represent different approaches used at schools, such as using a rights movement approach or rule oriented approach. To better understand differences between countries and schools regarding student voices practice, it might be important to go beyond written policies and aim to understand existing cultures and approaches available in different school settings.

About half (*n* = 7) of our 16 highly rated schools had openly published strategies related to anti-bullying or violence prevention and five schools reflected student diversity. In the wider dataset (412 documents) in six countries, student voices were most often identified in those describing anti-bullying or violence prevention programs (*n* = 75) or school’s mission statements (*n* = 72). Bullying in immigrant populations has been a subject of much research in recent years [51]. A study by Walsh, De Clercq, Molcho, Harel-Fisch, Davison, Madsen and Stevens [52] involving 10 European countries and the USA, found higher levels of physical fighting and bullying perpetration for both immigrant and native adolescents in schools with a higher percentage involving immigrant students. Consequently, the school climate and how it is perceived by the wider school community, impacts a student’s sense of safety and acceptance both in the classroom and within the wider school [53]. Anti-bullying guidelines should therefore be formulated and made publicly available for the whole school in joined initiatives with students, and visible to teachers, parents, and the wider community.

In our data, student diversity was reflected in less than a third of documents and only 45 inclusion or diversity strategies were found from websites of the 305 schools. We found this surprising as the schools were selected based on their location in areas with a high immigrant population. In general, the number of immigrant students has increased sharply over the past 20 years in most OECD countries with 13% of students on average having an immigrant background [54]. Research has shown that a minority of students might be, in reality, involved in decision-making at school [15]. This is especially problematic for students with immigrant backgrounds who might have to learn a new language and deal with discrimination as well as other obstacles related to their immigration experience [21].

Noteworthily, it remains unclear as to how immigrant students’ voice are sought in school decision-making processes. European countries differ regarding implementation of curricula for diverse student populations. In some countries, multi- and intercultural education is obligatory (in England) while in others it is mandatory (in Austria). Thus, it is up to the schools and perhaps teachers, on the extent they implement intercultural education in their lessons thus promoting the participation of minority groups. Therefore, schools and teachers play an important role as they can foster inclusion and multiculturalism by providing a learning environment that welcomes student diversity and its presence in the curriculum [55]. After understanding how hearing immigrant students’ voices in publicly available school documents is described, further studies are needed to explore the sense of belonging of immigrant students at school settings and how their voices are heard in diverse school environments from the viewpoint of immigrant students themselves including exploring their views of their teachers’ role in listening to their voices and viewpoints.

### Limitations

To date, little specific guidance is available to help researchers make the most of information regarding policy document analysis [33]. Therefore, our study has methodological limitations which need to be considered carefully. First, reviewers in six countries extracted and categorized data from school websites. Despite the inclusion and exclusion criteria, it is possible there were differences in understanding ‘school documents’ or documents complemented by other forms of student participation recommendations or unwritten values. This could lead to selection bias and impact the interpretations of the target documents. Second, since most policies are difficult to evaluate using controlled designs [31], the data in this study were analysed using a combination of qualitative and quantitative research methods. We carefully tested the categorization rules and data extraction tool (10 school websites in each country, totaling 60) prior to this study. Possible discrepancies in categorization processes were also discussed between reviewers to ensure congruence in the data extraction. However, subjective interpretations may still be evident in document analysis and the results may include biased interpretations. Third, there might be a fundamental difference between the actions of an individual organization and those of a public government with respect to several important factors such as reach, degree of compulsion, democratic legitimacy and use of public resources in each school [31]. It has also been reported that government-level policy documents related to students’ participation at school are more detailed and lengthy compared to local documents in different organizations [50]. These limitations may have affected our results, which may not be widely generalisable outside the European context.

Despite these limitations, we still believe that if the core values of the European educational systems, i.e. ethics, transparency, integrity, and mental wellbeing, are seen as important for schools, the evidence of student voices and their implementation would be found in relevant policy documentation publicly available to all. We also believe that schools publicly available documents serve as the main point of reference for teachers, students, leaders, and anybody interested in student voice initiatives as reflected by Gardiner and Ohi [56]. These outline the strategies and methods that should be employed to foster student voices and also represents the vision for implementing student voices in schools. By analysing these publicly available documents, we gained an understanding of how schools communicate their vision and identified ways to improve the integration of student voices into school policy documents. Therefore, school-based policies need to ensure that they are available to everyone to avoid social inequalities by involving all students including those from marginalized populations [31].

## Conclusion

Our document analysis based on publicly accessible school websites suggests that student voices are less frequently described in school written policy documents in our sample of European schools. Our findings provide a baseline to further monitor activities, not only at schools but also  to any public health authorities whose intention is to serve and openly share their values and practices in supporting students’ wellbeing with community members on their websites. Transparent, open, and visible policy documents could encourage students and their parents to join in decision-making to foster a feeling of responsibility, belonging and security. Through the methods utilised in this study, we can only conclude based on the extracted data, to what extent the topics are written into policy documents. Therefore, the next step is to collect empirical data using perceptions from students themselves on their school environment and the opportunities presented to them to show how student voices are realised in schools.

### Supplementary Information

**Supplementary Material 1.**

## Data Availability

The dataset used in this study is available from the corresponding author on reasonable request.

## Abbreviations

UNICEF: United Nations International Children's Emergency Fund
WHO: World Health Organisation
ID: Identification number
