# Supplementary material for: Engaging with immigrant students’ voices in the school environment: an analysis of policy documents through school websites
Source: BMC Public Health. 2024 Apr 19;24:1083. doi: 10.1186/s12889-024-18427-8 (PMC11027541; doi:10.1186/s12889-024-18427-8)
Supplement: Supplementary file 1 — Supplementary Material 1. [file 12889_2024_18427_MOESM1_ESM.docx]

**Additional file 1: A description of school system in study countries**

Austria: public schools are funded by the state, federal states, or municipalities. The schools follow the school teaching law [1], which regulates the internal order of the school system as the basis for cooperation between teachers, students, and legal guardians as a school community. Within this law the role of pupil representatives, parent`s club, school and class forums are defined. There are several other regulations and initiatives which determine participation of students. According to Austrian youth policy, student voice initiatives at schools should be implemented.

England: funding and management of schools is multiform. The most common school types are local authority run (primary schools) and comprehensive schools (secondary schools) who follow the National Curriculum. Foundation and voluntary schools have more freedom in their curricula. Academies and Free Schools are independent from the local authority and can follow a different curriculum, and Grammar schools are selective schools where students are selected based on ability tests. England also has a private school system which relies on fees paid by parents for their children’s education. At present there is no legislation requiring schools to implement student voice. However, the Education Act [2] states that schools must have regard to any guidance issued by the Secretary of State about consulting with students about decisions which affect them. Consequently, the D Department for Education [3] have issued the ‘Listening to and involving children and young people’ statutory guidance which applies to all local authority and maintained schools.

Finland: schools are maintained by municipalities and less than 2% of comprehensive schools are private or state schools. Finnish schools follow the national core curriculum for basic education that provides a framework for local curricula. Students’ active involvement at school is regulated by the Basic Education Act [4]. The act requires that all students should have the opportunity to participate in school action and development and to express their opinions about matters relating to them. Schools should also have student association activities.

Germany: the municipalities are usually in charge of public schools. At the national level, there is a joint declaration by the education ministers that student voices will be considered in school decisions on an institutional basis [5], but the educational system as a whole and thus also student participation rules at schools are legally regulated at the level of the federal states (e.g. SchulG NRW, 2016). This legislation recognizes the basic right of each student to participate. In practice, the focus is on student councils composed of the class representatives as well as school committees with elected student representatives.

Romania: the majority of schools are funded by the state although there are also private schools for all levels of education. The educational activities are guided by the Law of National Education (1/2011) and supervised by the Ministry of Education. There is no specific legislation about student participation, but a lot of activities take place. Students mainly participate through the formal institution called The Council of Students (active at both local schools and national level), and they can express their needs and demands, they can formulate issues and occasionally take part in decision-making processes.

In Switzerland, most children attend local schools run by local municipalities. Switzerland is culturally diverse with four official languages, and the federal structure delegates the authority for the school system to the 26 cantons, which hamper the coordination and joint development of national policies. There is no national legislation about student voice initiatives: Student participation on the level of classroom and schools has been described by [6] resulting in children expressing that they don’t really have a say.

**References**

1. **Bundesgesetz über die Ordnung von Unterricht und Erziehung in den im Schulorganisationsgesetz geregelten Schulen (Schulunterrichtsgesetz - SchUG)** [<https://www.ris.bka.gv.at/GeltendeFassung.wxe?Abfrage=Bundesnormen&Gesetzesnummer=10009600>]

2. **c.32** [<https://www.legislation.gov.uk/ukpga/2002/32/contents>]

3. Department for Education D: **Listening to and involving children and young people: Statutory guidance from the Department for Education**. In*.*; 2014.

4. **Basic Education Act 628/1998** [<https://www.finlex.fi/fi/laki/kaannokset/1998/en19980628.pdf>]

5. **Demokratie als ziel, gegenstand und praxis historisch-politischer bildung und erziehung in der schule: Beschluss der kultusministerkonferenz vom 06.03.2009 i. d. F. vom 11.10.2018** [<https://www.kmk.org/fileadmin/Dateien/pdf/PresseUndAktuelles/2018/Beschluss_Demokratieerziehung.pdf>]

6. Müller-Kuhn D, Häbig J, Zala-Mezö E, Strauss N-C, Herzig P: **"So richtig Einfluss auf den Unterricht haben wir nicht“ – Wie Schülerinnen und Schüler Partizipation wahrnehmen**. In: *Partizipation und Schule: Perspektiven auf Teilhabe und Mitbestimmung von Kindern und Jugendlichen.* edn. Edited by Gerhartz-Reiter S, Reisenauer C. Wiesbaden: Springer Fachmedien Wiesbaden; 2020: 187-206.
